# Synthetic biology: enormous possibility, exaggerated perils

**DOI:** 10.1186/1754-1611-2-7

**Published:** 2008-04-25

**Authors:** Zachary N Russ

**Affiliations:** 1 Fischell Department of Bioengineering, Room 2330 Jeong H. Kim Engineering Building (Bldg. #225), University of Maryland, College Park, MD 20742, USA

## Abstract

The following essay was written by a freshman undergraduate student majoring in Bioengineering at the University of Maryland, Mr. Zachary Russ. Mr. Russ was one of 94 students who submitted a 1000 to 1200 word essay to the 3rd Annual Bioethics Essay Contest sponsored by the Institute of Biological Engineering (IBE). A group of professionals in Biological Engineering assessed and ranked the essays in a blinded process. Five semi-finalists were invited to present their essays at a session at the annual meeting of IBE in Chapel Hill, NC on March 8, 2008. Five judges scored the presentations at the annual meeting and selected Mr. Russ's contribution as the overall winner (1st Place). Below is his essay.

## Introduction

Nobel Laureate and physicist Richard P. Feynman once wrote, "What I cannot create, I do not understand." In the field of biology, this quote is very apropos. Bioengineering, for all of its accomplishments, still has yet to create life to fit the same sort of precise specifications that chemical engineers have done with molecules and that mechanical engineers have with machines. Life is too unpredictable and poorly understood to reach its full engineering potential. The newly introduced field of synthetic biology (synbio) represents a response to that problem. Synbio is epitomized by the BioBricks project, which attempts to create life from scratch with clearly defined and understood interactions between each engineered gene. One success of the BioBricks approach is the complete rewrite of the T7 bacteriophage, which behaves much like the original, but in a very predictable fashion [1]. Synbio, much like genetic engineering in general, is not without its critics.

Many of the public's fears are reflected in *The Guardian *columnist Madeleine Bunting's assertion that "[s]cientists have a new way to reshape nature, but none can predict the cost [2]." Bunting writes that synbio "is a frightening science," that it "has the potential to be highly accessible ... making the task of regulating its use extremely difficult," and synthetic organisms can "get out and cross with their wild cousins, mutating into organisms we had never foreseen." She expresses concern over the "massive and momentous consequences," comparing synbio to the Industrial Revolution. Professional scientists' concerns are more limited to feasibility, suggesting that it is unlikely humans can design a better biological machine than nature can.

Many of these concerns stem from a misunderstanding of synbio. Synbio is a new approach to genetic engineering that knocks out of the genome the byproducts of evolution either by designing the organism from scratch or removing all of the nonessential DNA from an existing organism. The BioBricks project takes the former approach, and, by designing organisms using standardized building blocks, makes genetic engineering easier and much more efficient. BioBricks provide the potential to make biochemicals and pharmaceuticals quickly and easily without having to worry about unforeseen interactions between the drug genes and vestigial genes [3]. Life effectively becomes predictable and can be modeled by equations. All nonessential processes can be removed in synthetic organisms, so more energy and resources go towards making the product. In the process, engineers gain a greater understanding of life and its mechanisms.

But what of the drawbacks? What will happen if a synthetic organism escapes a lab? Most likely, nothing. With complete control over the genomes of their organisms, synbioengineers can introduce specific weaknesses into their organisms, such as a dependency on a particular amino acid, so that lab experiments have little chance of surviving in the wild. Without the noise data that natural organisms have in their genomes, synthetic organisms will also be very limited in genetic variation and virtually unable to adapt to new conditions. The probability of them hybridizing with wild varieties is also limited – they are synthetic organisms that have little in common with natural life and thus even less chance of recombinants surviving. Compared to organisms created using previous genetic engineering methods, synthetic organisms are much safer, not only because they lack the survival techniques found in nature, but also because they are better understood. Every gene in the organism is there intentionally with a specific purpose and a known function. This greatly lessens the risk of unknown genes triggering allergic reactions, one of the major fears in genetically modified food.

While accidental harm is significant, in the age of terrorism in which we live, malice is also an important factor in technologies. While the accessibility of BioBricks makes regulation difficult, synthetic biology is (and will probably continue to be) a long way from a basement technology. Those who would wish to visit destruction on the human race have much easier, more convenient tools available. Nature has already provided massive numbers of harmful organisms, and deadly chemicals are easier to manage and create. With so many variables, the human body is a far cry from the controlled laboratory environment that synthetic biology addresses. Scientists would have enormous difficulty in creating a virulent pathogen instead of a simple microbe designed to produce a chemical in a sterile, stable environment. Even the advent of computer programming provided an easy route to destruction, much easier than the slight advantage BioBricks provides, by allowing individual computer crackers to cause billions of dollars of damage with a few days of work, but few would argue against the information revolution for this threat.

There is even more risk to not using synthetic biology. Synthetic biology can produce organisms to clean up pollution and provide clean energy. Proteins and food can be created without wasting energy on a full-sized organism, providing cheap ways to make nutritious meals for millions. Without the help of synthetic biology, the Earth may just become uninhabitable for humans as pollutants build up and as the climate changes. The dangers posed to the human race are not from genetic engineering so much as they are from well-understood technologies and mechanisms – wasteful energy use, inefficient land use, and straightforward destruction. In many developing countries, respiratory problems stemming from pollution are a persistent and growing problem. Synthetic organisms hold the potential to manufacture products with minimal waste cleanly and efficiently, and some may even alleviate the threat of a poisonous atmosphere by breaking down specific toxins.

Ultimately, synthetic biology is about complete understanding. Synbio is the opportunity to gain power over life and achieve the ultimate goal in engineering – to be so familiar with a part that its behavior can be modeled, predicted, and used; and the uses are limitless. Already, a synbio project is close to producing large quantities of artemisinin, an antimalarial chemical [4]; other projects are aimed to make biosensors, anticancer microbes, and clean petrochemicals. Synbio is the next step in the growth of genetic engineering, and it offers a great hope for not only helping humanity but also minimizing the potential pitfalls of genetic engineering. Synthetic biology should be embraced, not feared, for the immense potential it has. As the MIT AI Lab's Tom Knight said, "the genetic code is 3.6 billion years old. It's time for a rewrite [5]."

## Authors' contributions

ZR wrote the essay and read and approved the overall manuscript.
